# Population-specific brain [^18^F]-FDG PET templates of Chinese subjects for statistical parametric mapping

**DOI:** 10.1038/s41597-021-01089-1

**Published:** 2021-11-26

**Authors:** Hongkai Wang, Yang Tian, Yang Liu, Zhaofeng Chen, Haoyu Zhai, Mingrui Zhuang, Nan Zhang, Yuanfang Jiang, Ya Gao, Hongbo Feng, Yanjun Zhang

**Affiliations:** 1 School of Biomedical Engineering, Faculty of Electronic Information and Electrical Engineering, Dalian University of Technology, Dalian, Liaoning China; 2 Department of Nuclear Medicine, the First Affiliated Hospital of Dalian Medical University, Dalian, Liaoning China

**Keywords:** Brain imaging, Radionuclide imaging

## Abstract

Statistical Parametric Mapping (SPM) is a computational approach for analysing functional brain images like Positron Emission Tomography (PET). When performing SPM analysis for different patient populations, brain PET template images representing population-specific brain morphometry and metabolism features are helpful. However, most currently available brain PET templates were constructed using the Caucasian data. To enrich the family of publicly available brain PET templates, we created Chinese-specific template images based on 116 [^18^F]-fluorodeoxyglucose ([^18^F]-FDG) PET images of normal participants. These images were warped into a common averaged space, in which the mean and standard deviation templates were both computed. We also developed the SPM analysis programmes to facilitate easy use of the templates. Our templates were validated through the SPM analysis of Alzheimer’s and Parkinson’s patient images. The resultant SPM t-maps accurately depicted the disease-related brain regions with abnormal [^18^F]-FDG uptake, proving the templates’ effectiveness in brain function impairment analysis.

## Abstract (summary)

**Table:** 

Measurement(s)	brain metabolism measurement
Technology Type(s)	FDG-Positron Emission Tomography
Factor Type(s)	age • sex
Sample Characteristic - Organism	Homo sapiens
Sample Characteristic - Location	China

Machine-accessible metadata file describing the reported data: 10.6084/m9.figshare.16382418

## Background & Summary

A series of studies in the last decade have reported that Positron Emission Tomography (PET) can be used as a clinical indicator of brain function impairments. As a functional imaging modality, PET reveals the metabolism level of molecular tracer in human brains. Typically, significant changes in PET tracer uptake are related to brain function abnormality. To detect the brain regions with abnormal PET tracer uptake, the patient images are compared with the reference images of normal participants. This comparison is performed using the Statistical Parametric Mapping (SPM) approach, which maps the patient PET image into a standard anatomical space and then conduct regional signal quantification and inter-subject comparisons. In this process, a template PET image representing healthy brain morphometry and PET tracer uptake level is required to provide the normal standard. The template image is generally constructed by registering and averaging a set of normal images. To guarantee the representativeness of the template, it is crucial to select the sample participants from the same population group of the target patient since there are significant differences in brain morphology and tracer metabolism between different population groups^1–4^.

Several well-known brain templates have been developed for different imaging modalities. Begin from the last century, the non-digital Talairach template^5^ played an important role in early neuroimaging studies. Later, the MNI-305 template^6,7^ was constructed based on the Talairach template and 305 3D Magnetic Resonance images. The ICBM-152 template^8–10^ was then developed using the MNI-305 template and higher resolution MRI images. These templates have been incorporated into several open-source tools such as SPM (http://www.fil.ion.ucl.ac.uk/spm/), the FMRIB’s Software Library (FSL, http://fsl.fmrib.ox.ac.uk/fsl/fslwiki/FSL) and commercial software tools such as PMOD (PMOD Technologies Ltd., Adliswil, Switzerland) and NeuroQ (Syntermed, Inc., Atlanta, GA, United States).

So far, most of the publicly available templates were constructed using Caucasian population data. For non-Caucasian populations, some templates of the MR modality have been developed (e.g., the Chinese-56^1^ atlas, the Chinese2020^11^ atlas_,_ the Brainnetome Atlas^12^ and Indian brain template^4^), but the existing PET templates are still based on the Caucasian population data^13^. To the scope of our knowledge, only one non-Caucasian [^18^F]-FDG template was reported in the Chinese literature but it was not open access^14^.

To enrich the family of publicly available brain PET templates, this study creates Chinese-specific brain PET templates based on [^18^F]-FDG PET images of 116 normal participants. Our templates have the following features:

- The templates were registered to the Chinese2020 MR atlas space which represents the standard brain morphometry of the Chinese population.
- The templates were constructed for the [^18^F]-FDG tracer widely used in clinical scenarios. The templates not only include the average PET image of the normal participants, but also the standard deviation image revealing the variation range of normal [^18^F]-FDG uptake.
- Along with the template data, we also provide the Matlab programmes for SPM analysis. The programme automatically maps the user’s patient images into the template space and compare the mapped patient image with the normal control group images. The SPM analysis results (e.g., t-maps) are automatically generated for abnormality assessment.

## Methods

**Fig. 1:**
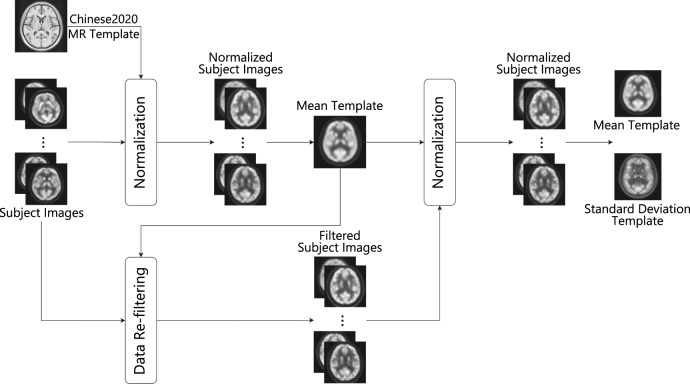
The workflow of brain template creation.

As shown in Fig. 1, the creation of the brain PET template follows a two-step procedure. Firstly, all the subject images are spatially mapped into a standard Chinese brain atlas space and their intensities are normalized to the same range. An initial mean template is created by averaging the normalized images and then a re-filtering step is performed to exclude the images with significant intensity difference to the mean template. After the re-filtering, the above normalization is performed again to create the final mean template and the standard deviation template. Details of the template creation are described in the following subsections.

### Dataset selection

In this retrospective study, experienced nuclear medicine doctors were invited to select the [^18^F]-FDG brain PET images of 119 participants without Alzheimer’s disease (AD), Parkinson’s disease (PD), diabetes, several psychiatric conditions or other abnormalities from the hospital database, according to the diagnose records of PET/CT scan, blood examination and behaviour tests. Table 1 reports the age and gender distributions of the selected participants. In subsequent steps, further data re-filtering will be performed based on the 119 images, and three of these images will be removed, leaving 116 images as the final sample set.

**Table 1:** Datasets.

Gender/Age	20–40	41–60	61–81
Male	16	36	25
Female	9	27	3

The PET images were acquired using the Biograph 64 PET/CT Scanner (SIEMENS, Germany) with 1.5 mm slice thickness, 336 × 336 × 110 image matrix dimension and 1.01821 mm pixel size. The participants fasted for 4–6 hours before the intravenous injection of 0.2 mCi/kg [^18^F]-FDG and lied rest for 60 minutes before the three minutes static brain PET/CT tomography imaging. The images were reconstructed using the Ordered Subset Expectation Maximization (OSEM) method with corrections for attenuation, scattering, stopping time and scan normalization. Statical PET/CT images were eventually obtained, and only PET images were selected for this work. This retrospective study was approved by our institutional review board and complied with the ethical committee standards. Facial features of the participants were removed from the PET images by setting the values of all the extracerebral voxels to zero.

### Spatial normalization

To eliminate the inter-subject morphological differences, all the images were mapped into a standard brain atlas space. We used the Chinese2020 template^11^ constructed from over 2000 Chinese MRI images as the standard atlas to represent Chinese brain anatomy. A non-rigid three-dimensional (3D) image registration method based on smooth diffeomorphic spatial transform (named Symmetric Normalization, SyN) was applied to align each subject PET image with the Chinese2020 template^15^. The SyN method uses mutual information as the similarity metric for cross-modality registration^16^, it results in a smooth symmetric diffeomorphic mapping from the subject space to the atlas space, preventing the generation of excessive local warping and anatomically unrealistic deformation. The SyN method was implemented using the ANTsPy toolbox^17^ with a gradient step size of 0.2 and a field smoothing variance equal to three times the voxel spacing.

### Intensity normalization

Considering that the subject PET images may have inconsistent pixel intensities ranges due to image acquisition procedure variation, intensity normalization was performed to ensure comparable intensity ranges of all the subject images. In the literature, there are several ways of intensity normalization, such as dividing pixel values by the mean value of a reference brain region or mapping a certain percentage range of the global maximum intensity to [0, 1]^18^. We adopted a popular method which divided the image intensity by the mean value of the pixels within 40–90% of the maximum brain voxel intensity^19^. For further flexibility, if the users want to apply other intensity normalization methods, they can download our original PET images shared in the data repository for their own normalization.

### Template creation

After spatial and intensity normalizations, the mean and standard deviation (std.) template images were obtained by computing voxel-wise mean and std. values from all the participants. Considering that these templates will be used with the popular SPM toolbox for data analysis, we smooth the mean and std. templates with an isotropic 3D Gaussian kernel of 8 mm FWHM to match the template image resolution suggested by the SPM toolbox^13^.

### Data re-filtering

To ensure the health normality of our dataset, a data re-filtering step was applied after the template creation. The image-level Sum of Squared Differences (SSD)^20,21^ was calculated between each normalized subject and the mean template to screen the subject with potential metabolic abnormalities. The SSD of subject *k* was calculated as $SS{D}_{k}={\sum }_{i}{\left({I}_{k}\left(i\right)-{I}_{mean}\left(i\right)\right)}^{2}$, where *I*_*k*_ is the normalized image of subject *k*, *I*_*mean*_ is the mean template image and *i* is the voxel index. The suspected anomalous subject was removed from the dataset based on the three-sigma rule. Let *SSD*_*mean*_ and *SSD*_*std*_ be the mean and std. of the SSDs of all the participants, subject *k* is excluded from the dataset if $\left|SS{D}_{k}-SS{D}_{mean}\right| > 3\cdot SS{D}_{std}$. Based on this rule, three potential abnormal participants were removed and then the mean and std. templates were computed again using the 116 remaining subject images.

### SPM analysis

To facilitate easy use of our template data, we also provided the Matlab programme of SPM analysis using our templates. We implemented the General Linear Model (GLM) method for SPM analysis by calling the widely used SnPM toolbox^22^. The programme registers the user’s test images to our mean template image for spatial normalization. It also applies intensity normalization to the test images using the same method used for template construction. Afterwards, the user’s images are compared with our normal control group images using the voxel-wise non-parametric two-sample t-test. In the t-test, an adjustable family-wise error (FWE) significance level threshold was used to distinguish the abnormal pixels in the patient image, and the resultant t-map can be plotted for visual inspection.

## Data Records

All the 116 subject images, the mean and std. templates and the Matlab programme for SPM analysis have been shared on the NITRC website^23^ (10.25790/bml0cm.95). We saved all the images in the popular NIfTI format used by the neuroimaging research community. All skulls will be stripped from the images due to privacy concerns with the participants.

## Technical Validation

### The template images

**Fig. 2:**
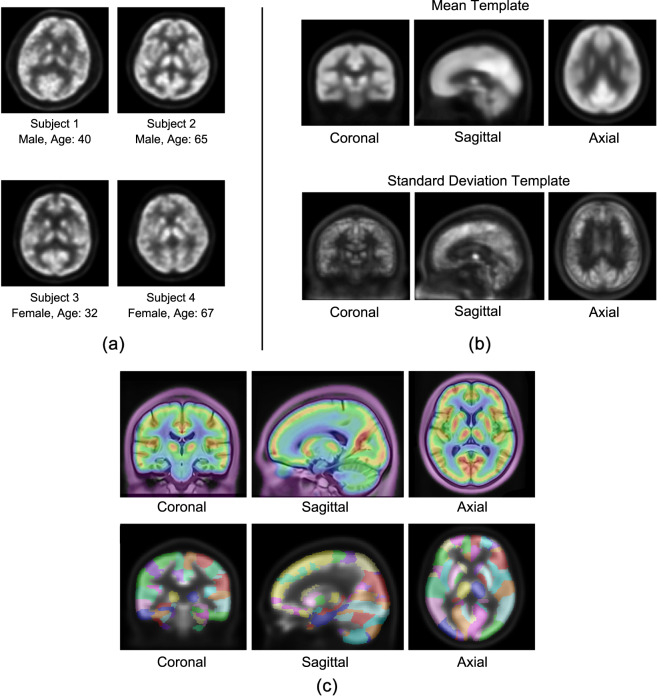
Representative subject images and the constructed template images. (**a**) Four representative subject images of different ages and genders. (**b**) The mean template and the standard deviation template. (**c**) The fusion of the mean PET template and the Chinese2020 MR template (the first row) and the brain region labels of Chinese2020 (the second row).

The selected image set and the constructed templates are shown in Fig. 2. Figure 2(a) demonstrates four representative subject images of different genders and ages. Figure 2(b) shows the mean template and std. template created from the selected images. The mean template clearly illustrates the [^18^F]-FDG distribution in the brain and the std. template represents the inter-subject metabolic variances. As the std. template shows, the frontal region has relatively larger variances implying that the participants have more metabolic differences in this area. The combination of the mean and std. templates can be used to calculated voxel-wise z-score values of the user’s patient image. Figure 2(c) demonstrates the fusion view of our mean template and the Chinese2020 MR template, providing combined anatomical and functional reference information for Chinese neuroimage analysis. Figure 2(c) also shows the fusion view of the mean PET template and the brain region labels provided by the Chinese2020 template. These labels can be used for computing the regional uptake in sub-brain structures.

### Template validation

**Fig. 3:**
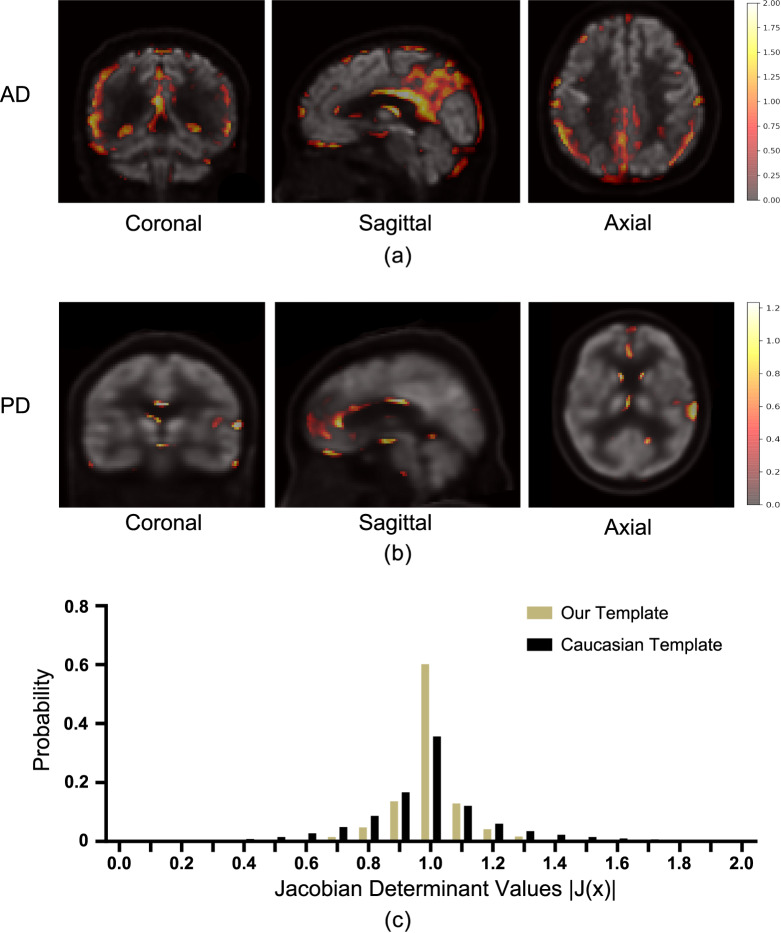
SPM analysis results (t-maps) of (**a**) AD and (**b**) PD patient images. The colour-coded areas indicate the locations where the voxel values of the patient are significantly different from the normal control group (FWE>0.05). The brightness of the colour represents the t-value. (**c**) Comparison of the voxel |**J**(*x*)| distributions resulted from our template and the Caucasian template.

Degenerative brain diseases (e.g., AD and PD) are usually accompanied by abnormal [^18^F]-FDG metabolism in certain brain regions. Our template data and normal control group images provide the normal range of [^18^F]-FDG metabolism corresponding to each voxel location in the brain, helping the user to identify regions with abnormal metabolism. To test the reliability of our template for patient data analysis, we conducted an SPM analysis of the AD and PD patients PET images. Retrospective images of the patients were collected from the hospital database and the diseases were diagnosed according to clinical imaging, behaviour and experimental tests. Each single patient image was registered to the mean template and compared to the normal control group images using our Matlab programme and the resultant t-maps are shown in Fig. 3(a,b). The colour-coded areas in the figures indicate the locations where the voxel values of the patient are significantly different from the normal control group (FWE > 0.05). The brightness of the colour represents the t-value. It can be observed from Fig. 3(a) that the t-map of the AD patient has abnormal voxels in the temporal, parietal lobes and posterior corpus callosum, as well as parts of the frontal cortex and cingulate gyrus which are the main regions affected by AD^24,25^. Similarly, in Fig. 3(b), regional metabolic abnormalities can be seen in the basal ganglia and the temporal lobes of the PD patient. This result coincides well with the pathological characteristics of PD, i.e., cell death in the brain’s basal ganglia is manifested on [^18^F]-FDG PET as reduced metabolism in the frontal and parietotemporal^26^.

Moreover, to compare our Chinese-specific template with the Caucasian PET template, we registered each of the 116 original PET images to both our template and the Caucasian template of the SPM software. The registration produced deformation vector fields which warped the individual images to the template space. Following the image registration literature^27^, we computed the Jacobian matrix determinant |**J**| of the deformation field to quantify the extent of anatomical distortion caused by the registration. For each voxel *x* in the individual image, the Jacobian matrix determinant |**J**(*x*)| denotes the ratio of local volume change at the position of *x*, e.g., |**J**(*x*)| = 1 means isometric transform, |**J**(*x*)| = 0.9 indicates 10% volume shrinking and |**J**(*x*)| = 1.1 means 10% volume expansion. If the template has a similar brain shape to the individual image, the warping from individual to template should be close to isometric, thus |**J**(*x*)| should be close to 1 for most voxels. Therefore, by observing the probability distribution of |**J**(*x*)| of all the voxels in the 116 images, we can assess the extent of anatomical distortion induced by the anatomical differences between the individual image and the template. Figure 3(c) compares the |**J**(*x*)| distributions resulted from our template and the Caucasian template. Our template resulted in a distribution more centralized to 1 than the Caucasian template. The Caucasian template has a |**J**(*x*)| range between 0.4 and 1.8, implying notable local volume shrinking and expansion caused by the inter-population anatomical differences. In contrast, our template results in less extent of distortion because we adopted the average Chinese brain shape from the Chinese2020 atlas.

## Data Availability

The code of patient image SPM analysis using our template data has been uploaded to both the NITRC repository^23^ (https://www.nitrc.org/projects/cnpet/) and our group’s GitHub website (https://github.com/DlutMedimgGroup/Chinese-Brain-PET-Template) along with the template data. In detail, the code includes the steps of patient image spatial normalization, intensity normalization and the two-sample t-test.
